# Resolvin E1 accelerates pulp repair by regulating inflammation and stimulating dentin regeneration in dental pulp stem cells

**DOI:** 10.1186/s13287-021-02141-y

**Published:** 2021-01-22

**Authors:** Jie Chen, Huaxing Xu, Kun Xia, Shuhua Cheng, Qi Zhang

**Affiliations:** Department of Endodontics, School & Hospital of Stomatology, Tongji University, Shanghai Engineering Research Center of Tooth Restoration and Regeneration, 399 Middle Yan Chang Road, Shanghai, 200072 China

**Keywords:** Resolvin E1 (RvE1), Human dental pulp stem cells (hDPSCs), Inflammation, Pulp repair, Pulp regeneration

## Abstract

**Background:**

Unresolved inflammation and tissue destruction are considered to underlie the failure of dental pulp repair. As key mediators of the injury response, dental pulp stem cells (DPSCs) play a critical role in pulp tissue repair and regeneration. Resolvin E1 (RvE1), a major dietary omega-3 polyunsaturated fatty-acid metabolite, is effective in resolving inflammation and activating wound healing. However, whether RvE1 facilitates injured pulp-tissue repair and regeneration through timely resolution of inflammation and rapid mobilization of DPSCs is unknown. Therefore, we established a pulp injury model and investigated the effects of RvE1 on DPSC-mediated inflammation resolution and injured pulp repair.

**Methods:**

A pulp injury model was established using 8-week-old Sprague-Dawley rats. Animals were sacrificed on days 1, 3, 7, 14, 21, and 28 after pulp capping with a collagen sponge immersed in PBS with RvE1 or PBS. Hematoxylin-eosin and Masson’s trichrome staining, immunohistochemistry, and immunohistofluorescence were used to evaluate the prohealing properties of RvE1. hDPSCs were incubated with lipopolysaccharide (LPS) to induce an inflammatory response, and the expression of inflammatory factors after RvE1 application was measured. Effects of RvE1 on hDPSC proliferation, chemotaxis, and odontogenic differentiation were evaluated by CCK-8 assay, transwell assay, alkaline phosphatase (ALP) staining, alizarin red staining, and quantitative PCR, and possible signaling pathways were explored using western blotting.

**Results:**

In vivo, RvE1 reduced the necrosis rate of damaged pulp and preserved more vital pulps, and promoted injured pulp repair and reparative dentin formation. Further, it enhanced dentin matrix protein 1 and dentin sialoprotein expression and accelerated pulp inflammation resolution by suppressing TNF-α and IL-1β expression. RvE1 enhanced the recruitment of CD146^+^ and CD105^+^ DPSCs to the damaged molar pulp mesenchyme. Isolated primary cells exhibited the mesenchymal stem cell immunophenotype and differentiation. RvE1 promoted hDPSC proliferation and chemotaxis. RvE1 significantly attenuated pro-inflammatory cytokine (TNF-α, IL-1β, and IL-6) release and enhanced ALP activity, nodule mineralization, and especially, expression of the odontogenesis-related genes *DMP1*, *DSPP*, and *BSP* in LPS-stimulated DPSCs. RvE1 regulated AKT, ERK, and rS6 phosphorylation in LPS-stimulated DPSCs.

**Conclusions:**

RvE1 promotes pulp inflammation resolution and dentin regeneration and positively influences the proliferation, chemotaxis, and differentiation of LPS-stimulated hDPSCs. This response is, at least partially, dependent on AKT, ERK, and rS6-associated signaling in the inflammatory microenvironment. RvE1 has promising application potential in regenerative endodontics.

**Supplementary Information:**

The online version contains supplementary material available at 10.1186/s13287-021-02141-y.

## Background

The existence of mesenchymal stem cell populations in adult tissues that conduce tissue cell turnover and respond to tissue damage is, in general, accepted [1]. In dental pulp tissue, dental pulp stem cells (DPSCs), besides their active effects in self-renewal and multipotential differentiation, also regulate pulp repair and regeneration by suppressing the immune response and modulating the secretion of inflammatory factors [2–4]. Dental pulp tissue injury caused by dental caries and trauma is generally characterized by an immune response and inflammatory cell infiltration. The outcome of pulp injury is determined by the balance between inflammation and regeneration [5]. Excess or prolonged inflammatory responses have a very destructive effect on vital pulp and eventually lead to total tissue necrosis, whereas moderate inflammation can promote tissue regeneration by initiating DPSC migration, proliferation, and differentiation [6–8]. Therefore, inflammation should be resolved in a timely manner and the self-repair capacity of the dental pulp should be enhanced to preserve the remaining vital pulp and repair the damaged pulp.

Inflammation resolution has long been considered to be a passive process. However, recent studies have shown that it is an active, programmed process controlled by endogenous specialized pro-resolution lipid mediators (SPMs), including resolvins, lipoxins, protectins, and maresins [9, 10]. These pro-resolution mediators are actively enhanced in the early pathological stage to modulate the inflammatory response; however, their expression diminishes in the advanced disease stage [11]. This implies that inflammation may become excessive and sustained when these mediators are dysregulated. Therefore, exogenous application of endogenous mediators to prevent pulp inflammation from developing into the irreversible stage and rescue the unstable microenvironment has received great research interest.

Resolvin E1 (RvE1), one of the SPMs, is of particular interest because of its outstanding anti-inflammatory and pro-resolution effects in various inflammatory disease models. RvE1 has been shown to directly act on tendon stromal cells to promote inflammation resolution in periarticular tendinitis [12], to accelerate corneal epithelial cell migration in corneal inflammatory injury [13], and to improve myofibroblast proliferation to suppress renal interstitial fibrosis in a renal injury model [14]. In rat molar damaged pulp tissue, topical application of RvE1 limited inflammatory cell infiltration [15, 16]. Our previous studies investigated the effects of RvE1 on dental pulp fibroblasts (DPFs) during the pathogenesis of pulpitis and proved its potential in inhibiting pulp inflammation and promoting resolution by suppressing DPF activation [17]. Moreover, dental-derived stem cells (e.g., periodontal ligament stem cells, stem cells from the apical papilla) have been found to produce and release SPMs, which generate a favorable microenvironment for dental stem cells to promote tissue self-repair [18, 19]. However, whether RvE1 can promote the repair and regeneration of dental pulp damage by regulating inflammation resolution and mobilizing DPSCs remains unclear.

Therefore, we established a rat pulp injury model to investigate the effects of RvE1 on DPSC-mediated inflammation resolution and injured pulp repair. We observed that RvE1 promoted pulp repair by resolving pulp inflammation, forming reparative dentin, and enhancing the ability of DPSCs to proliferate, chemotax, and differentiate, at least in part by regulating the phosphorylation of AKT, ERK, and ribosomal protein S6 (rS6) in LPS-stimulated DPSCs.

## Materials and methods

### Ethics statement

All experimental animal studies and the use of human pulp cells were approved by the Institutional Review Board of Tongji University (approval number: SL2018R5). The experimental procedures abided by ethics standards, and written informed consent was obtained from the volunteers.

### Pulp injury model establishment

The pulp injury model was established as previously described [20, 21]. Eight-week-old male Sprague-Dawley rats (*n* = 60) were used [22]. The bilateral first molars in the maxillary were used, respectively. The animals were anesthetized by intraperitoneal injection of 30 mg/kg pentobarbital sodium (Sigma-Aldrich, St. Louis, MO, USA). Then, a half-moon cavity was created with a No.1/4 round steel bur (Shofu, San Marcos, CA, USA) in the mesial half of the occlusal surface of the upper first molar. When red pulp was visible at the bottom, we used the tip of a sterile dental explorer to expose the pulp. The entrance of the pulp horn was enlarged with K-files #40 to a length of 1 mm and a diameter of 1 mm. An aseptic cotton ball was pressed into the cavity to secure hemostasis. After removal of the cotton ball, PBS containing 50 ng RvE1 (Cayman Chemical, Ann Arbor, MI, USA) or PBS alone as a control was injected into the exposure site, a collagen sponge (JinLing Pharma, Nanjing, China) was covered, and 1 mm BP plus (Innovative Bioceramix, CA, USA) was placed on the pulp-exposed cavity. All cavities were immediately restored with composite resin (Kerr Corp, Orange, CA, USA). The doses of RvE1 were based upon previous dose–response studies in rat [15, 17, 23]. On days 1, 3, 7, 14, 21, or 28 after the operation, the animals in the treated and non-treated (control) groups were sacrificed by intracardiac perfusion with 4% paraformaldehyde (PFA) buffered at pH 7.2–7.4.

### Sample collection and histological analysis

The samples were immersed in 4% PFA at 4 °C for 24 h, then demineralized by immersing in a 10% EDTA solution at 4 °C for approximately 2 months, with a solution change every 3 days. The samples were dehydrated using a graded ethanol series, embedded in paraffin, and sectioned at 4-μm thickness. After deparaffinization and rehydration, the sections were stained with hematoxylin and eosin (Sangon Biotech, Shanghai, China) and Masson’s trichrome (Nanjing Jiancheng Bioengineering Institute, Nanjing, China). We determined the porosity of the dentin bridge by measuring the percentage of the spaces containing cells within the reparative dentin using ImageJ [24]. Images of the reparative dentin were captured using a microscope (Nikon Eclipse 80i, Japan). We marked the boundary of the reparative dentin and used ImageJ to measure its area.

For immunohistochemical staining, the sections were processed with hyaluronidase and goat serum blocking (MXB, Fuzhou, China). Next, they were incubated with primary antibody at 4 °C overnight. The primary antibodies included anti-DMP1 (1:200; gift from Dr. Chunlin Qin, TA&M University, College of Dentistry), anti-DSP (1:300; GeneTex, USA), anti-ChemR23 (1:200; Abcam, USA), anti-BLT1 (1:200; Boster, Wuhan, China), anti-TNF-α (1:200; Proteintech, USA), and anti-IL-1β (1:200; Affinity Biosciences, USA). After washing in PBS, the sections were incubated with biotinylated secondary antibodies and further processed using streptavidin peroxidase and a DAB Detection Kit (MXB, Fuzhou, China) per the manufacturer’s instructions. Positively stained areas were measured with ImageJ.

For immunofluorescence staining, the sections were subjected to antigen retrieval and treated with goat serum (MXB, Fuzhou, China). Then, they were incubated with anti-CD105 (1:200; Abcam), anti-CD146 (1:200; Abcam), anti-ChemR23 (1:200; Abcam), and anti-BLT1 (1:200; Boster) at 4 °C overnight. After washing with PBS, the sections were incubated with fluorescence-labeled secondary antibodies (1:1000; Invitrogen, USA) for 1 h. The sections were counterstained with DAPI and observed under a fluorescence microscope (Nikon Eclipse 80i, Japan).

### Human (h) DPSC isolation, culture, and characterization

Human dental pulp tissue was isolated from normal impacted third mandibular molars or normal premolars for orthodontic therapy from healthy patients (13–20 years old) with informed consent at Tong Ji Stomatology Hospital. We also obtained written parental consent for the minors before the study was begun. Briefly, dental pulp tissue was collected, cut into pieces, and digested with 0.3% collagenase for 30 min to obtain a single-cell suspension. The cells were cultured in minimal essential medium alpha modification (α-MEM; Hyclone, USA) containing 10% fetal bovine serum (FBS; ExCell Bio), 100 U/mL penicillin, and 100 mg/mL streptomycin at 37 °C in 5% CO_2_. Cell clones were isolated and expanded. Stemness surface markers and pluripotency were evaluated as described previously [25]. In brief, the hDPSC phenotype was determined by flow cytometry using the following antibodies: anti-CD34-PE (BD Biosciences), anti-CD45-PE (R&D systems), anti-CD105-APC (BD Biosciences), anti-CD146-FITC (R&D Systems), and anti-CD90-PE (R&D Systems).

Proliferation ability was assessed by a colony-forming unit (CFU) assay. hDPSCs were seeded at 200 cells/well in 6-well plates in growth medium and incubated at 37 °C in 5% CO_2_. After 14 days, the cells were fixed with 4% PFA, stained with 0.5% crystal violet, washed with distilled water, and dried. Aggregates of 50 cells or more were scored as 1 CFU.

For the multilineage differentiation assay, alizarin red S, alcian blue, and oil red O staining were used to identify osteogenic, chondrogenic, and adipogenic differentiation, respectively.

### Proliferation and Transwell assays

For the cell proliferation assay, 2 × 10^3^ cells/well were seeded in 96-well plates (Corning, NY, USA) in α-MEM containing 10% FBS and incubated overnight at 37 °C in 5% CO_2_. Then, the cells were exposed to 1, 10, 50, 100, or 200 nM RvE1 or control (PBS). After incubation for 1, 3, 5, or 7 days, CCK-8 was added to each well and the plates were further incubated for 2 h. Then, the optical density at 450 nm was read using a microplate reader (Bio-Tek, Hercules, CA, USA).

The chemotaxis ability of hDPSCs treated with RvE1 or PBS for 24 h or 48 h was assessed in 24-well plates, using Transwell Filter Inserts (Corning). hDPSCs (2 × 10^4^ cells) in 200 μL of 5% FBS/α-MEM were seeded on the cell culture inserts. In the lower chambers, 750 μL of 5% FBS/α-MEM containing RvE1 (10, 100, or 200 nM) or control (PBS) was added. After incubation for 24 h or 48 h, the cells on the upper side of the insert were scraped off a using cotton-tipped swab. The cells that had passed through the insert membrane were fixed with 4% PFA and stained with 0.5% crystal violet. Cells were analyzed in four random fields under an inverted microscope (Nikon Eclipse 80i).

After comprehensively analyzing the results, the concentration of 100 nM RvE1 was selected for further experiments.

### Alkaline phosphatase (ALP) activity assay and alizarin red S staining

hDPSCs were seeded in 24-well plates (Corning) and cultured in odontoblastic differentiation medium containing 2 mM β-glycerophosphate (Sigma-Aldrich), 50 mg/mL ascorbic acid (Sigma-Aldrich), and 10^− 7^ M dexamethasone (Sigma-Aldrich) and supplemented with either 1 μg/mL lipopolysaccharide (LPS), 100 nM RvE1, 1 μg/mL LPS + 100 nM RvE1, or the control (PBS). The medium was changed every 2 days. After 1 and 2 weeks, the medium was removed for ALP staining. The cells were fixed in in 4% PFA for 20 min. After the cells were rinsed with PBS three times, 5-bromo-4-chloro-3-indolyl phosphate/nitroblue tetrazolium solution (Beyotime, Shanghai, China) was added to each well for 10 min. Then, the cells were washed and photographed. For quantitative analysis, 10% (w/v) cetylpyridinium chloride solution (Sigma-Aldrich) was added for 30 min, and the staining intensity was quantified by measuring the absorbance at 562 nm in a microplate reader (Bio-Tek).

Mineral deposits in the cultured cells were stained with alizarin red to evaluate odontoblastic differentiation. After 2 and 3 weeks, cells were fixed with 4% PFA and stained with 0.2% alizarin red (Sigma-Aldrich) at room temperature for 20 min. The alizarin red S-positive area was analyzed under a stereoscopic microscope (Zeiss, Germany). For quantitative analysis, we used 10% (w/v) cetylpyridinium chloride solution and measured the optical density at 450 nm in a microplate reader (Bio-Tek).

### Assessment of inflammation- and differentiation-related gene expression in response to LPS treatment by quantitative reverse-transcription (RT-q) PCR

To evaluate the capability of RvE1 in countering inflammation, hDPSCs were pretreated with LPS (1 μg/mL) to induce an inflammatory condition. Then, 100 nM RvE1 was added and the cells were cultivated for 1, 3, or 7 days to determine mRNA levels of *TNF-α*, *IL-1β*, and *IL-6*, for 7, 14, and 21 days to determine mRNA levels of dentin matrix protein1 (*DMP1*), dentin sialophosphoprotein (*DSPP*), and bone sialoprotein (*BSP*). Total RNA was isolated using Trizol reagent. RNA was reverse-transcribed using a Transcriptor First-Stand cDNA Synthesis Kit (Roche, Schlieren, Switzerland). qPCRs were run using the FastStart Essential DNA Green Master Kit (Roche) and a LightCycler 96 Instrument (Roche). The details of the protocols were previously described [26]. The primer sequences (Sango Biotech, Shanghai, China) are listed in Table 1.

**Table 1:** Specific primer sequences for quantitative RT-PCR

Gene	Forward primer	Reverse primer
*GAPDH*	GGAGCGAGATCCCTCCAAAAT	GGCTGTTGTCATACTTCTCATGG
*IL-1β*	TGGCTTATTACAGTGGCAATGAGGATG	TGTAGTGGTGGTCGGAGATTCGTAG
*IL-6*	GGTGTTGCCTGCTGCCTTCC	GTTCTGAAGAGGTGAGTGGCTGTC
*TNF-α*	CGTGGAGCTGGCCGAGGAG	AGGAAGGAGAAGAGGCTGAGGAAC
*DSPP*	AAAGTGGTGTCCTGGTGCAT	CCTGGATGCCATTTGCTGTG
*DMP1*	TTCCTCTTTGAGAACATCAACCTG	ACTCACTGCTCTCCAAGGGT
*BSP*	CACTGGAGCCAATGCAGAAGA	TGGTGGGGTTGTAGGTTCAAA
*ChemR23*	GGTGATAGGGGTGTTCCAGC	ATCACCAGACCATTGCCCAG
*BLT1*	TTCAGTTCTAGCGTCAACCCG	GCAACCAGCCAGTCCAAAAC

### Western blotting

hDPSCs (2 × 10^5^) were cultured in 6-well plates in α-MEM for 24 h. Then, the cells were starved overnight in α-MEM plus 1% FBS, gently washed with PBS, exposed to LPS for 30 min, and treated with RvE1 for 15, 30, or 60 min. Thereafter, the hDPSCs were lysed in radio immunoprecipitation buffer supplemented with 1% protease inhibitor cocktail and 1% phosphatase inhibitor cocktail (all from Beyotime Biotechnology, Nanjing, China). The cells were collected by scraping, lysed, and centrifuged at 8000×*g* for 20 min. The total protein concentration was measured using a BCA Protein Assay Kit (CWBiotech, China). Proteins (20 μg) were resolved using a 10% PAGE Fast Gel Preparation Kit (Shanghai EpiZyme Biotechnology, China) and transferred to a nitrocellulose membrane (Millipore, USA). After blocking in TBST (Tris Buffered Saline Tween) containing 5% nonfat milk, the membranes were incubated with primary antibodies against pAkt, Akt, pERK1/2, ERK1/2, prS6, rS6 (1:1000; Cell signaling, Danvers, MA, USA) overnight. α-Tubulin (1:1000; Abcam) was used as an endogenous control. Next, the membranes were washed thrice with TBST, incubated with horseradish peroxidase-conjugated secondary antibody (1:2000; CWBiotech) for 1 h, and washed thrice with TBST. Then, the membranes were treated with chemiluminescence reagent (Sigma-Aldrich) and visualized by enhanced chemiluminescence. Protein bands were quantified using ImageJ. The assay was performed three times using different samples.

### Statistical analyses

All experiments were performed at least in triplicate. Data are presented as the mean ± standard error of the mean. Differences between groups were analyzed by two-way analysis of variance (ANOVA) in SPSS 20.0 (IBM Corp, Armonk, NY, USA). *P* < 0.05 was considered statistically significant.

## Results

### RvE1 reduces the necrosis rate and preserves more vital pulp of damaged dental pulp

In the control group, many samples suffered from serious inflammation and were ending up with necrosis (see Additional file 1, Figure S1). The data we present in this article is relatively healed well in all control groups. In fact, the proportion of these well-healed control samples is quite small. Large proportion is the necrotic samples. In contrast, the results of the RvE1 group were relatively stable. About 70–80% of the samples are repair in the RvE1 group, while in the control group, only 50% of the samples are repair at 1 week, only 30% of the samples are repair at 4 weeks (see Additional file 1, Table.S1). And in the RvE1 group, the inflammation was resolved and the necrosis area was reduced gradually, while in the control group, the inflammation was processed fast and eventually lead to most of tissue necrosis (see Additional file 1, Table.S2). Therefore, in this article, the control group has significantly more necrotic samples than the RvE1 group. This is the biggest difference. We did not compare this, but compared the remaining healing samples in the control group with the RvE1 group. In addition, at 21 and 28 days in the control group, the continuity of the pulp tissue is interrupted, while in the RvE1 group, there are more vital pulp tissue between the newly formed dentin bridge and the bottom of pulp chamber and the pulp is continuous (see Additional file 1, Figure S2).

### RvE1 enhances the injured dental pulp repair and the generation of dentine-pulp complex in rats

**Fig. 1:**
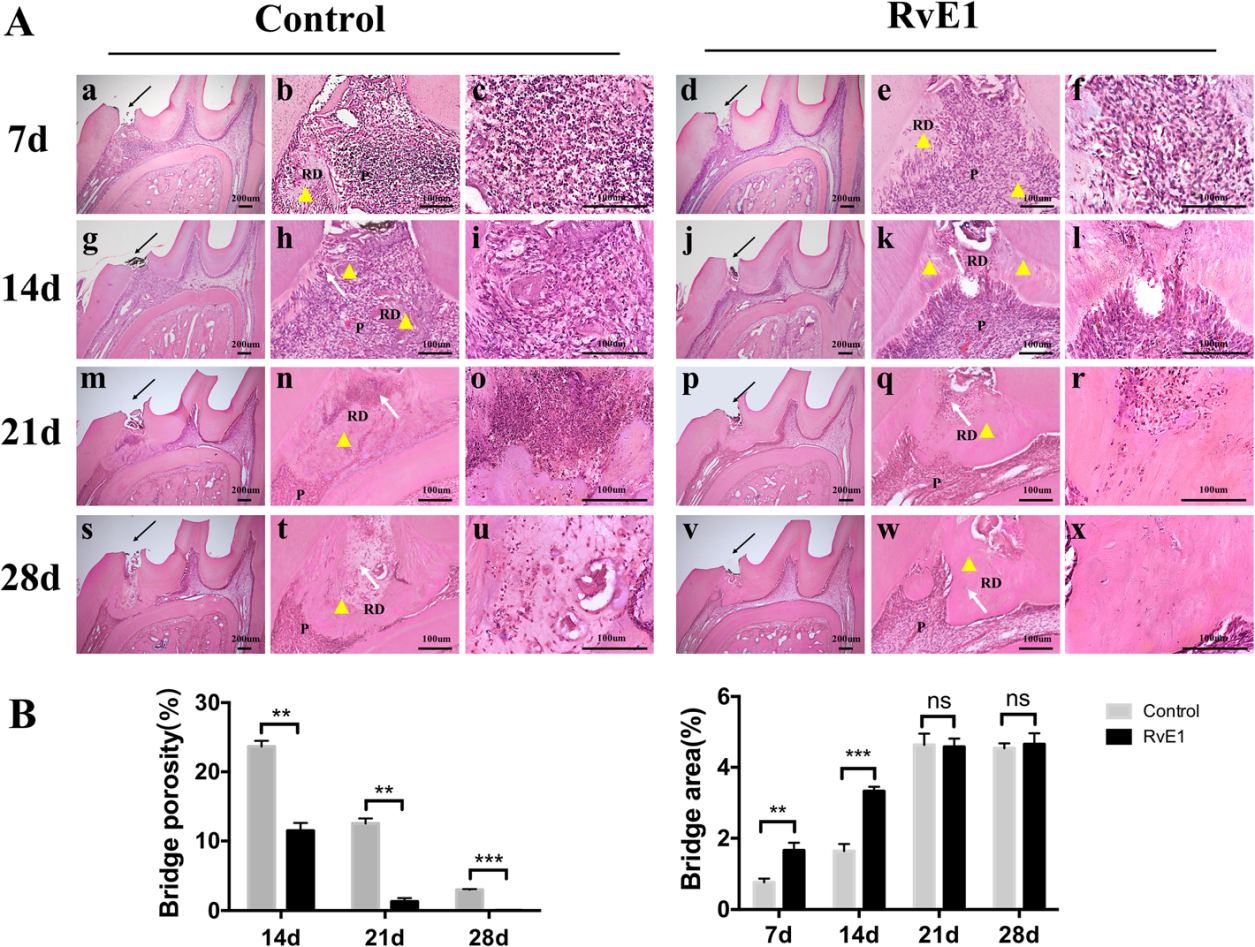
The effects of RvE1 on the dental pulp repair and the generation of dentine-pulp complex. **A** The HE staining results of the control group and the RvE1 application group. **b**, **e**, **h**, **k**, **n**, **q**, **t**, **w** Higher magnification views of the black arrow areas in panels **a**, **d**, **g**, **j**, **m**, **p**, **s**, and **v**. **c**, **f**, **i**, **l**, **o**, **r**, **u**, **x** Higher magnification views of **b**, **e**, **h**, **k**, **n**, **q**, **t**, and **w**, respectively. P pulp, RD reparative dentin. **B** Quantification of dentin bridge porosity, area of reparative dentin. The yellow triangle represents the reparative dentin. The black arrow represents the exposure site. The white arrow represents the cell inclusions. *P < 0.05, **P < 0.01, ***P < 0.001, *n* = 20

One week after treatment of pulp injury model rats with RvE1, mild inflammatory infiltrate was observed (Fig. 1A, d, e, f), whereas in the control group, intense inflammatory infiltrates and tissue disorganization were observed (Fig. 1A, a, b, c). After 2 weeks, reparative dentin was clearly observed at the exposure site in the RvE1 group, although the new dentin bridge appeared heterogeneous, with cell inclusions (Fig. 1A, j, k, l). In contrast, several diffuse calcifications were observed in the control group (Fig. 1A, g, h, i). At 3 weeks, newly formed dentin bridges with homogeneous and continuous structures began to close the exposure site in the RvE1 group (Fig. 1A, p, q, r), whereas in the control group, the reparative tissue was discontinuous and heterogeneous, with cell inclusions (Fig. 1A, m, n, o), and bacteria may ingress into the pulp through these porosities. At 4 weeks, odontoblast-like cells around the newly formed dentin bridges exhibited a polarized morphology and were arranged in a palisade layer in the RvE1 group, with continuous reparative bridges and well-distinguishable dentin tubules that contained a substantial amount of vital pulp tissue (Fig. 1A, v, w, x). In contrast, poorly organized reparative structures that closed the pulp chamber and prevented pulp continuity were observed in the control group (Fig. 1A, s, t, u). Semi-automatic image analysis showed that the porosity of the newly formed dentin bridge was higher in the control group than that in the RvE1 group, the dentin bridge in the RvE1 group was denser. The area of dentin bridge in the RvE1 group was larger than that in the control group at 1 and 2 weeks (*P* < 0.05) (Fig. 1b).

### RvE1 improves dentin mineralization ability in the damaged pulp repair process

**Fig. 2:**
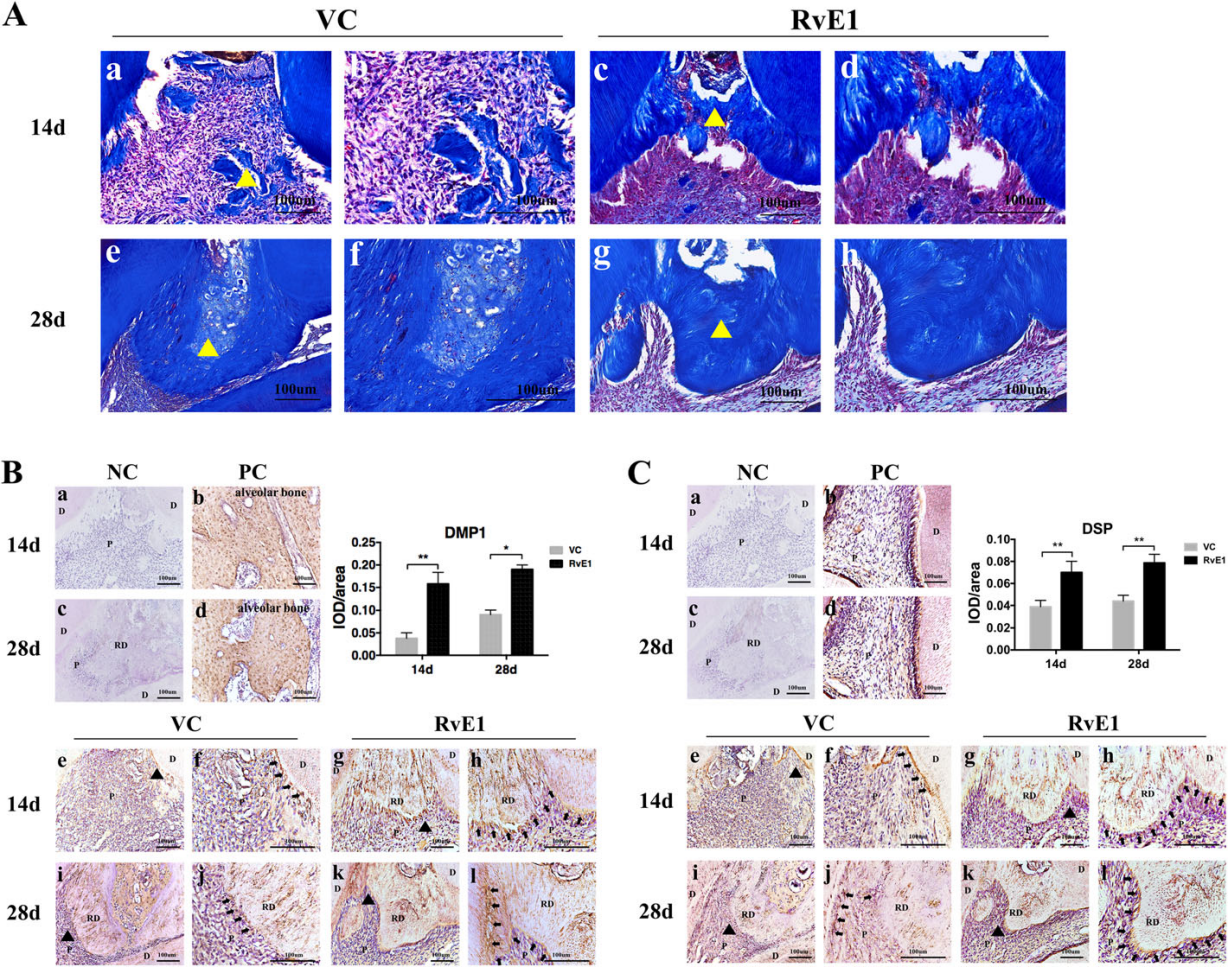
The evaluation of RvE1 on the dentin mineralization ability in the damaged pulp repair process. The Masson trichrome staining (**A**), the immunohistochemical staining and the quantification of DMP1 (**B**), and DSP (**C**) in the control group and the RvE1 application group. The collagen shows up in blue and celluloses in red. The yellow triangle represents the reparative dentin, and **b**, **d**, **f**, **h** higher magnification views of **a**, **c**, **e**, **g** (**A**). **f**, **h**, **j**, **l** Higher magnification views of the black triangle areas in panels **e**, **g**, **i**, **k**, and black arrows (**f**, **h**, **j**, **l**) indicate the positive signal, respectively (**B**, **C**). Right panel shows the quantitative measurements of integrated optical density (IOD)/area of DMP1 (**B**) and DSP (**C**). NC negative control, PC positive control, VC vehicle control, P pulp, D dentin, RD reparative dentin. *P < 0.05, **P < 0.01, ***P < 0.001, *n* = 10

In mature dentin, collagen fiber plays crucial roles in the mineralization of dentin matrix and the process of forming reparative dentin [27]. Masson’s trichrome-stained sections from samples obtained at 2 weeks showed that in the control group, several diffuse calcifications and loose and crooked rows of collagen fiber were observed (Fig. 2A, a, b), whereas collagen fiber showed neat rows in reparative dentin bridges that were formed in the RvE1 group (Fig. 2A, c, d). After 4 weeks, the control group still displayed hypocalcification, non-uniform dyeing (Fig. 2A, e, f), whereas the dentin bridge had a uniform tubular structure in the RvE1 group (Fig. 2A, g, h).

Dentin matrix protein 1 (DMP1) and dentin sialoprotein (DSP) were labeled as markers of active odontoblasts [28]. At 2 weeks, the RvE1 group showed remarkably higher expression of DMP1 (Fig. 2B, g, h) and DSP (Fig. 2C, g, h) in dentin matrix and the odontoblasts, whereas only the cells around the opening site were showed strong expression of DMP1 (Fig. 2B, e, f) and DSP (Fig. 2C, e, f) in the control group. Similar observations were made at 4 weeks. Especially, cells around the dentin bridge showed a polarized morphology and stronger DMP1, DSP expression of the dentin matrix and the odontoblasts in the RvE1 group (Fig. 2B, k, l) (Fig. 2C, k, l) as compared with the control group (Fig. 2B, i, j) (Fig. 2C, i, j). The quantitative analysis shows that RvE1 enhanced DMP1 and DSP expression (Fig. 2B, C).

### RvE1 accelerates inflammation resolution in rat damaged pulp tissue

**Fig. 3:**
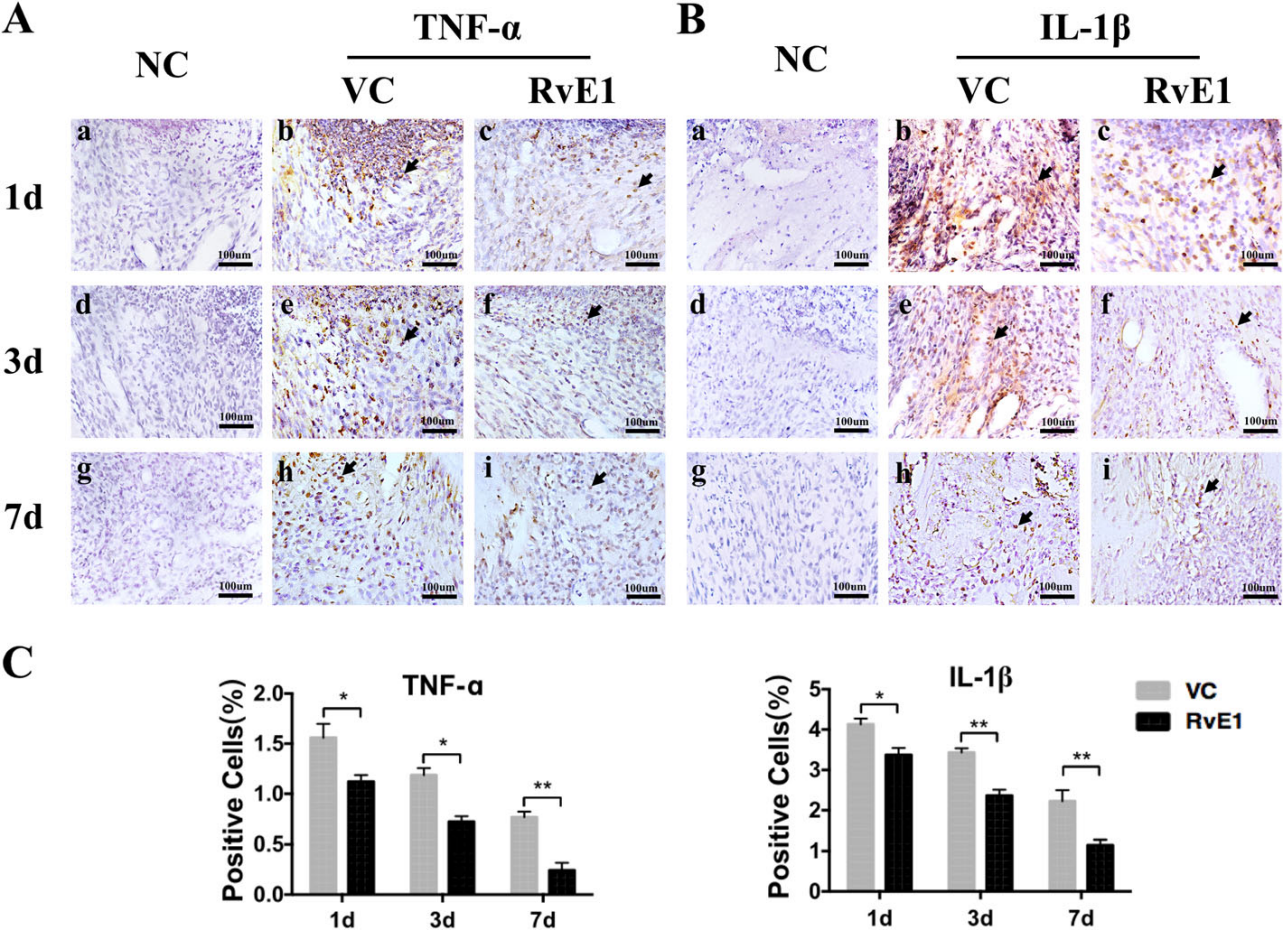
The effects of RvE1 on the resolution of pulp inflammation. Longitudinal follow-up of TNF-α (**A**) and IL-1β (**B**) expression by immunohistochemistry. Arrowhead indicates the positive cells. **C** Quantification of TNF-α and IL-1β. NC negative control, VC vehicle control. *P < 0.05, **P < 0.01, ***P < 0.001, *n* = 15

On day 1, both groups had intense inflammatory infiltrates. Polymorphonuclear cells were observed in the coronal portion of the teeth in the control group, whereas they were restricted to the exposure site in the RvE1 group. TNF-α-positive and IL-1β-positive cells were more evidently detected by immunohistochemistry in the control group (Fig. 3A, b) (Fig. 3B, b) than in the RvE1 group (Fig. 3A, c) (Fig. 3B, c). Three days after model establishment and treatment, TNF-α-positive cell infiltrates started to diminish in both groups (Fig. 3A, e, f). IL-1β-positive cells also decreased in both group (Fig. 3B, e, f). On day 7, TNF-α expression was strongly reduced in both groups (Fig. 3A, h, i), but more significantly so in the RvE1 group (Fig. 3A, i). IL-1β-positive cells were reduced in the RvE1 group (Fig. 3B, i) and more so than in the control group (Fig. 3B, h). Based on immunostaining quantification, we found that RvE1 showed a remarkable efficacy in reducing pulp inflammation by downregulating pro-inflammatory cytokines and suppressing inflammatory infiltrates (*P* < 0.05) (Fig. 3C).

### RvE1 enhances dental pulp stem cell commitment in the dental mesenchyme

**Fig. 4:**
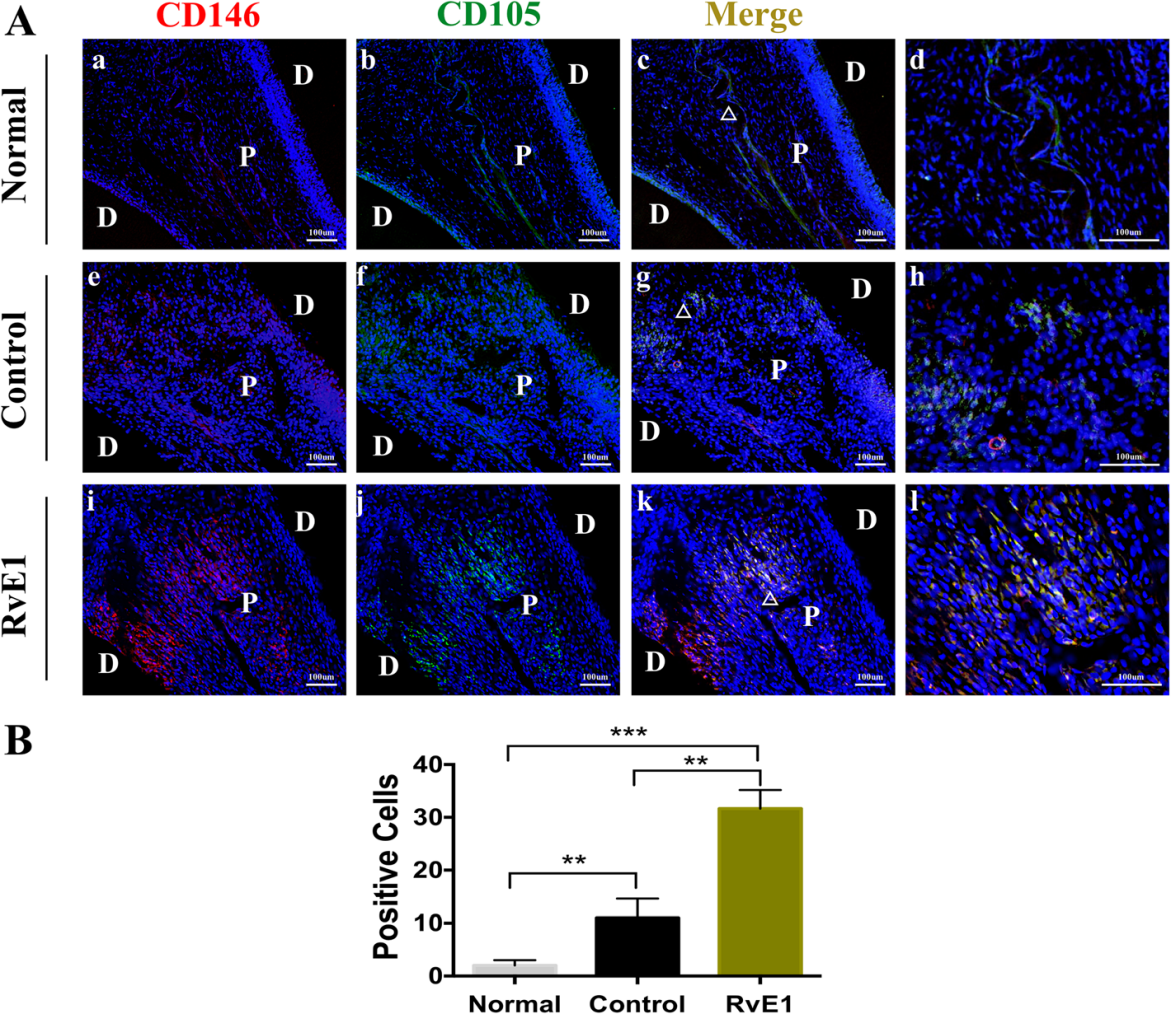
The effects of RvE1 on stem cell commitment in the dental mesenchyme. The immunofluorescence staining of CD146 (red), CD105 (green), and DAPI (blue) in the normal, the control, and the RvE1 application group (**A**). **d**, **h**, **l** Higher magnification views of white triangle areas in panels **e**, **g**, and **k**, respectively. D dental, P pulp. **B** Quantification of the positive cells. **P < 0.01, ***P < 0.001, *n* = 5

The tooth pulp is the source of cells that form reparative odontoblasts, and genetic lineage tracing of cells after extensive dentine damage in mice has shown that perivascular cells (pericytes) in the damaged site are stimulated to proliferate, leave the vessels, and differentiate into odontoblast-like cells that produce a reparative form of dentine to form a dentine bridge [29]. These pericytes express typical Mesenchymal stem cell (MSC) markers, including CD146, CD105, and Sca1. In the normal group without any treatment, CD146^+^ and CD105^+^ cells were mainly detected around the vasculature (Fig. 4A, a, b, c, d). Upon injury, CD146^+^ and CD105^+^ cells were detectable in the molar coronal pulp mesenchyme of control (Fig. 4A, e, f, g, h) and RvE1 groups (Fig. 4A, i, j, k, l). Immunostaining quantification revealed that these positive cells were more numerous in the RvE1 group than in the control group (*P* < 0.05) (Fig. 4B).

### Phenotype and characterization of hDPSCs

**Fig. 5:**
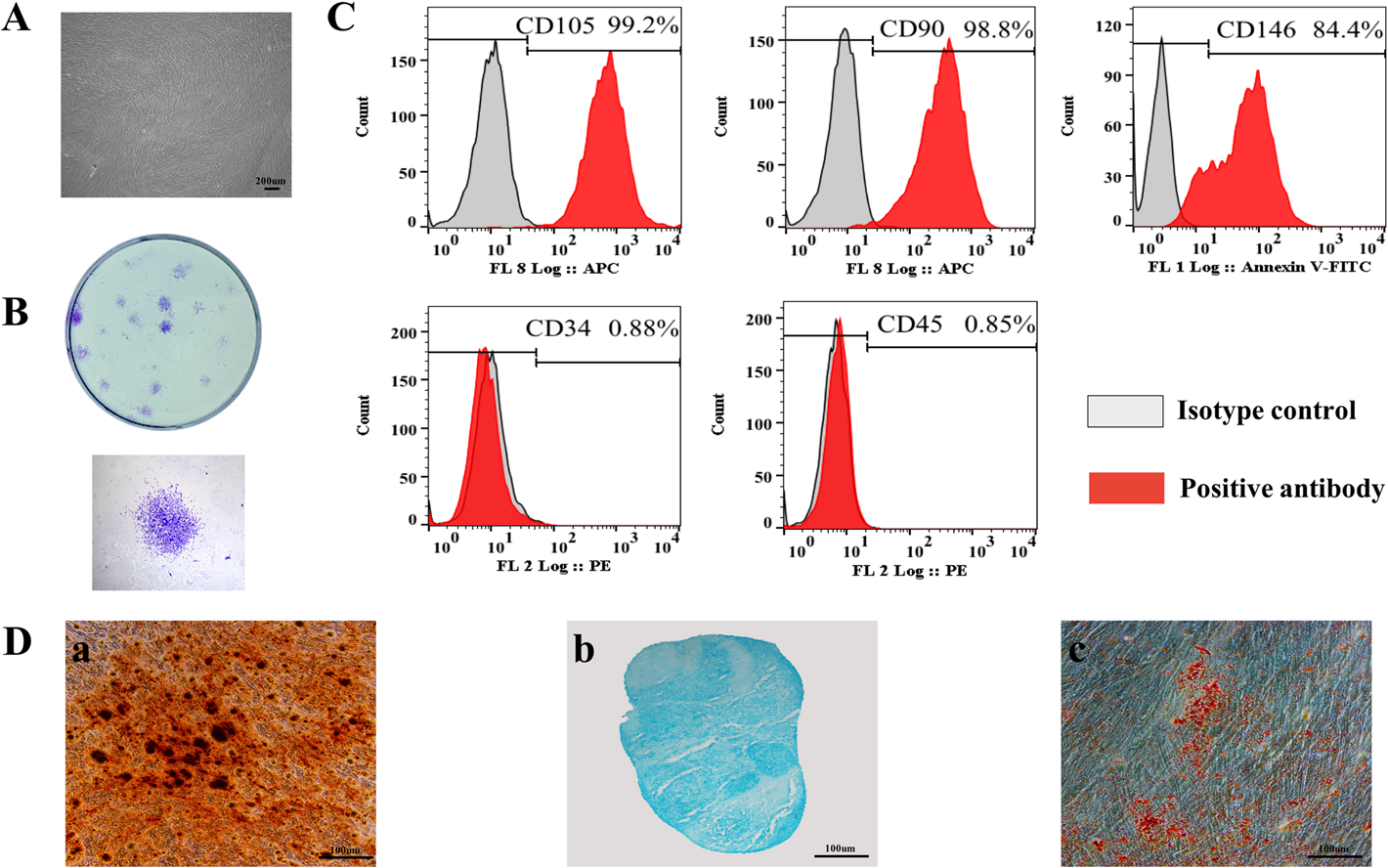
HDPSC immunophenotype and differentiation. Light microscopy image of passage-2 hDPSCs (**A**). Representative images of hDPSC colony-forming units at 14 days (**B**). Flow cytometric analysis of mesenchymal stem antigens in hDPSCs indicates they strongly express CD105, CD90, and CD146, but negative for CD34 and CD45 (**C**). The multilineage differentiation capacity of hDPSCs (**D**). Alizarin red staining of mineralization nodule formation (**a**). Alcian bale staining of chondrogenic induction (**b**). Oil red staining of adipogenic induction (**c**)

Primary cells presented a typical homogeneous spindle morphology (Fig. 5A) and were capable of producing colonies from single cells after 14 days of culture (Fig. 5B). hDPSCs expressed specific mesenchymal stem cell antigens, including CD105, CD90, and CD146. They stained negative for the hematopoietic markers CD34 and CD45 (Fig. 5C). Osteogenic, chondrogenic, and adipogenic differentiation capacities of the hDPSCs were determined at 3 weeks after induction. Mineralized nodule formation was observed after alizarin red staining (Fig. 5D, a), deposition of chondrogenic-like matrix was revealed by alcian blue staining (Fig. 5D, b), and lipid-droplet accumulation within cells was observed after oil red O staining (Fig. 5D, c), indicating that the hDPSCs were capable of differentiating into the respective tissues.

### RvE1 promotes the proliferation and chemotaxis of hDPSCs

**Fig. 6:**
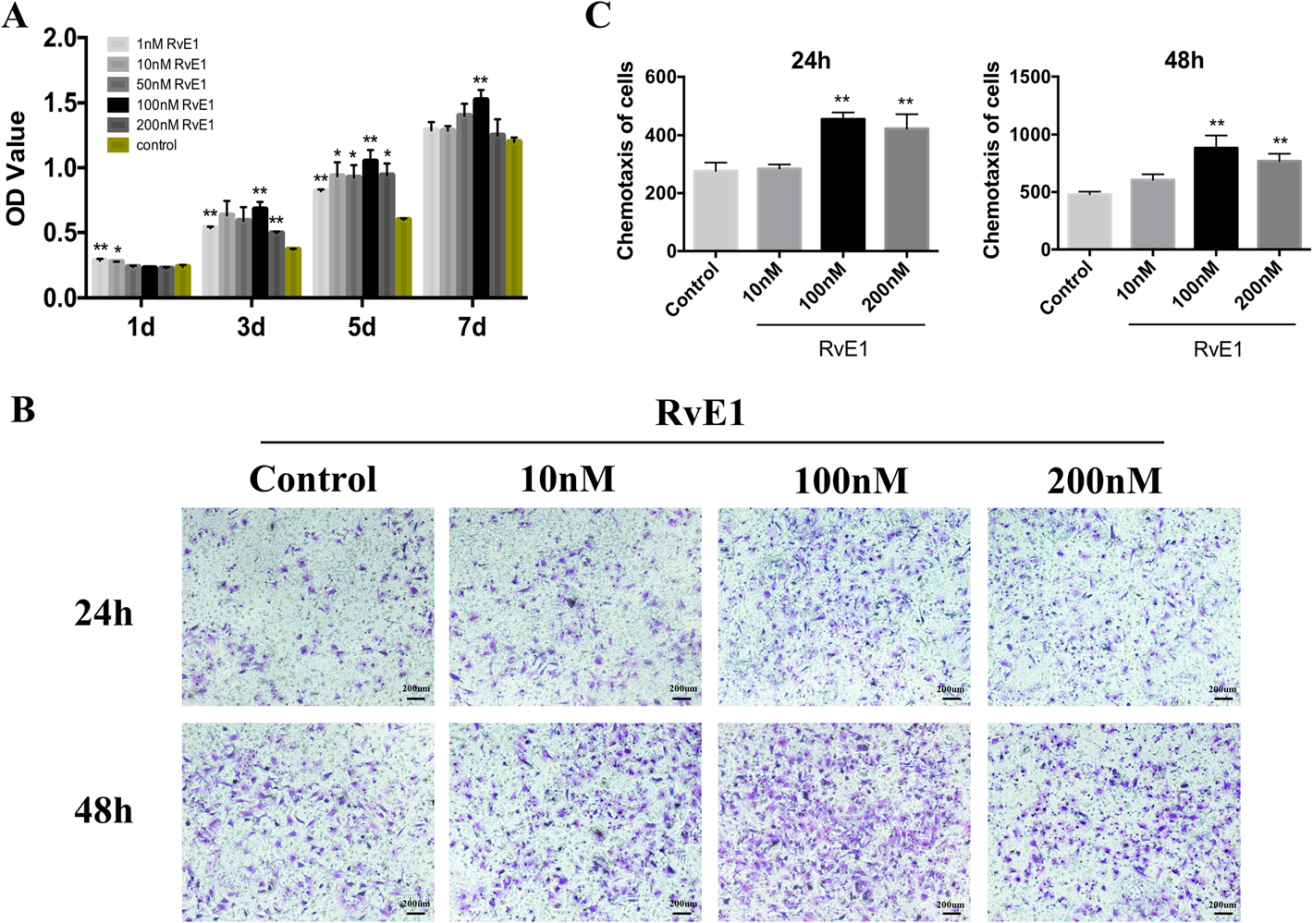
The effects of RvE1 on cell proliferation, chemotaxis of hDPSCs. Proliferation assessment of hDPSCs after culture with a series of concentration of RvE1 (1, 10, 50, 100, 200 nM) (**A**). The crystal violet staining of migratory cells from different groups after culture in RvE1 for 24 h and 48 h (**B**). Statistical analysis of the average migratory cell numbers per field from different groups (**C**). *P < 0.05, **P < 0.01, ***P < 0.001. The results are mean ± standard deviation of triplicate measurements from three independent experiments

A CCK-8 assay was used to evaluate the effect of RvE1 on hDPSC proliferation. Cells were incubated with RvE1 (1, 10, 50, 100, 200 nM) or PBS (control) for 1, 3, 5, or 7 days. On day 1, the proliferation ability unapparent increased after treatment with RvE1, whereas on 3, 5, and 7 days, the proliferation increased markedly after treatment with RvE1 as compared with the control, especially at 100 nM (Fig. 6A). A Transwell assay was used to assess the effect of RvE1 on hDPSC chemotaxis. Migratory cells in the RvE1 group were significantly more numerous, especially at 100 nM, than those in the control group after 24 and 48 h (Fig. 6B, C). Based on these findings, 100 nM RvE1 was used in all subsequent experiments.

### RvE1 suppresses inflammation-related genes in LPS-induced hDPSCs

**Fig. 7:**
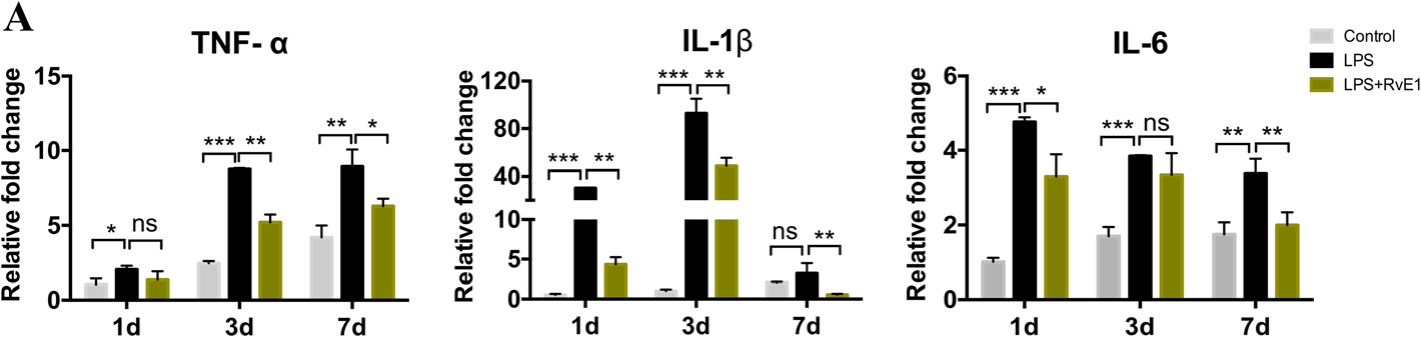
The effects of RvE1 on the expression of pro-inflammatory cytokines in LPS-stimulated hDPSCs. Cells were cultured with or without RvE1 in the presence of 1 μg/mL LPS for 1, 3, and 7 days. The release of TNF-α, IL-1β, and IL-6 were determined by qPCR. *P < 0.05, **P < 0.01, ***P < 0.001. The results are mean ± standard deviation of triplicate measurements from three independent experiments

hDPSCs were pretreated with 1 μg/mL LPS and then cultured in the presence or absence of RvE1 for 1, 3, or 7 days. Treatment with LPS increased the expression of pro-inflammatory cytokine genes (*TNF-α*, *IL-1β*, and *IL-6*) as determined by RT-qPCR (Fig. 7A). RvE1 significantly attenuated the expression of *TNF-α* on days 3 and 7. *IL-1β* expression in LPS-stimulated hDPSCs was significantly suppressed by RvE1 on days 1, 3, and 7. *IL-6* expression was strongly decreased on days 1 and 7 in LPS-stimulated hDPSCs by RvE1.

### RvE1 improves the odontogenic ability in LPS-stimulated hDPSCs

To assess the effects of RvE1 on the odontoblastic ability of LPS-induced hDPSCs, cells cultured in osteogenic medium (OM) were treated or not with 100 nM RvE1 in the presence of 1 μg/mL LPS for 7 and 14 days for ALP analysis, 14 and 21 days for alizarin red staining, and 7, 14, and 21 days for RT-qPCR analysis.

**Fig. 8:**
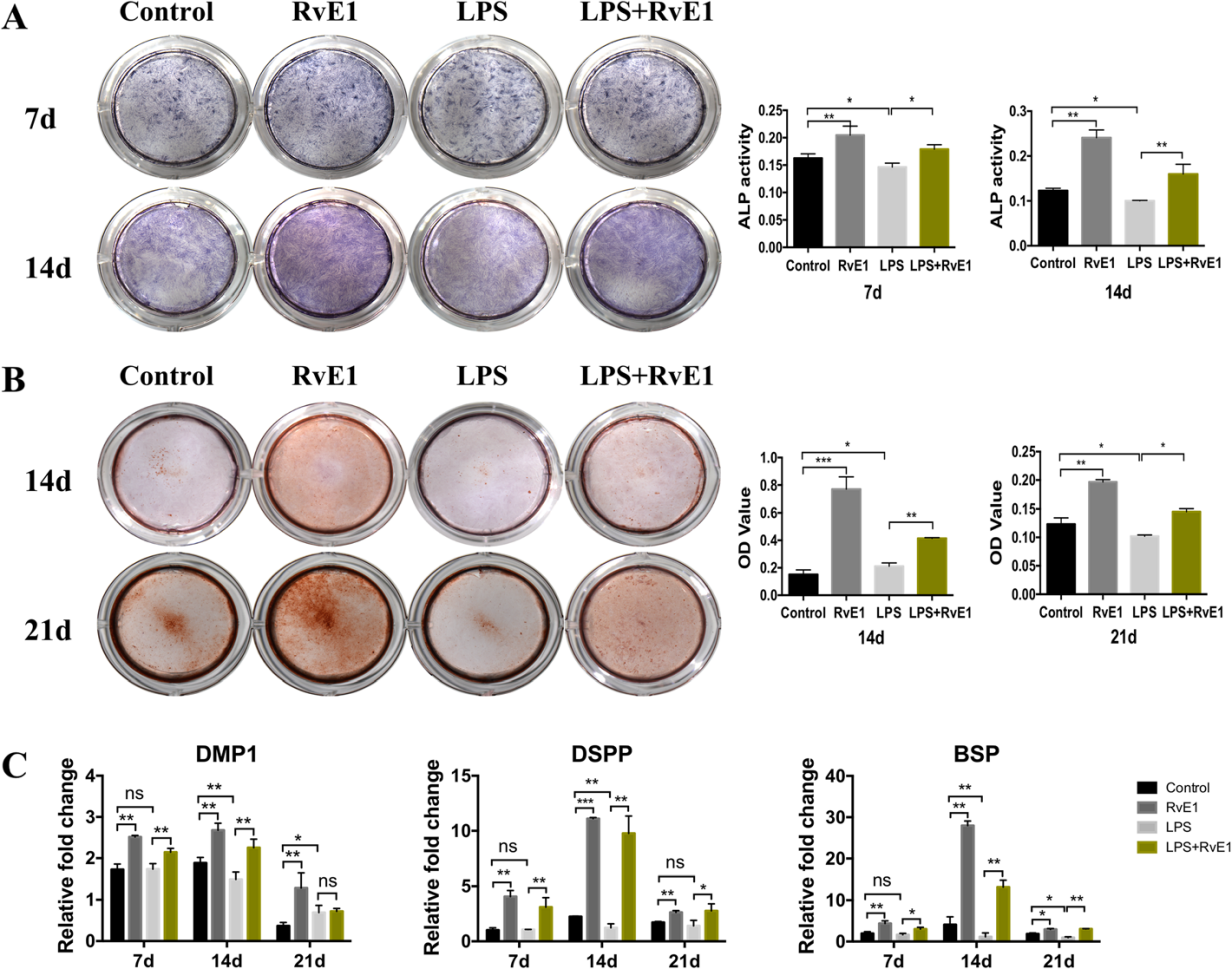
The effects of RvE1 on odonto/osteogenic differentiation in LPS-stimulated hDPSCs. Staining and quantitative detection of ALP activity in hDPSCs under incubation of RvE1 with or without 1 μg/mL LPS treated for 7 and 14 days (**A**). Alizarin red staining of the hDPSCs after culture in RvE1 with or without 1 μg/mL LPS treated for 14 and 21 days (**B**). Relative gene expression levels of DMP1, DSPP, and BSP of hDPSCs after culture in RvE1 with or without 1 μg/mL LPS treated for 7, 14, and 21 days (**C**). Control, PBS with osteogenic-induced medium group; LPS, 1 μg/mL LPS with the osteogenic-induced medium group; RvE1, 100 nM RvE1 with the osteogenic-induced medium group; LPS + RvE1, 1 μg/mL LPS and 100 nM RvE1 with the osteogenic-induced medium group. *P < 0.05, **P < 0.01, ***P < 0.001. The results are mean ± standard deviation of triplicate measurements from three independent experiments

On days 7 and 14, RvE1 enhanced the ALP activity of hDPSCs as compared with the activity level in the negative control (OM) group (*P* < 0.05) (Fig. 8A). LPS suppressed the ALP activity of hDPScs. RvE1 significantly promoted the ALP activity of LPS-stimulated hDPSCs as compared with that in the LPS groups (*P* < 0.05) (Fig. 8A). Alizarin red staining showed that the mineralized areas were significantly increased in the RvE1, LPS, and LPS + RvE1 groups as compared with the control group on day 14, and the staining intensity increased in the RvE1 and LPS + RvE1 groups but decreased in the LPS group on day 21 (*P* < 0.05) (Fig. 8B). mRNA levels of *DMP1*, *DSPP*, and *BSP* were significantly enhanced by RvE1 on days 7, 14, and 21. Under LPS-stimulated condition, RvE1 also enhanced the mRNA levels of *DMP1*, *DSPP*, and *BSP* as compared to those in the LPS group on days 7, 14, and 21 (*P* < 0.05) (Fig. 8C).

### RvE1 activates the PI3K-Akt and ERK signaling pathways

**Fig. 9:**
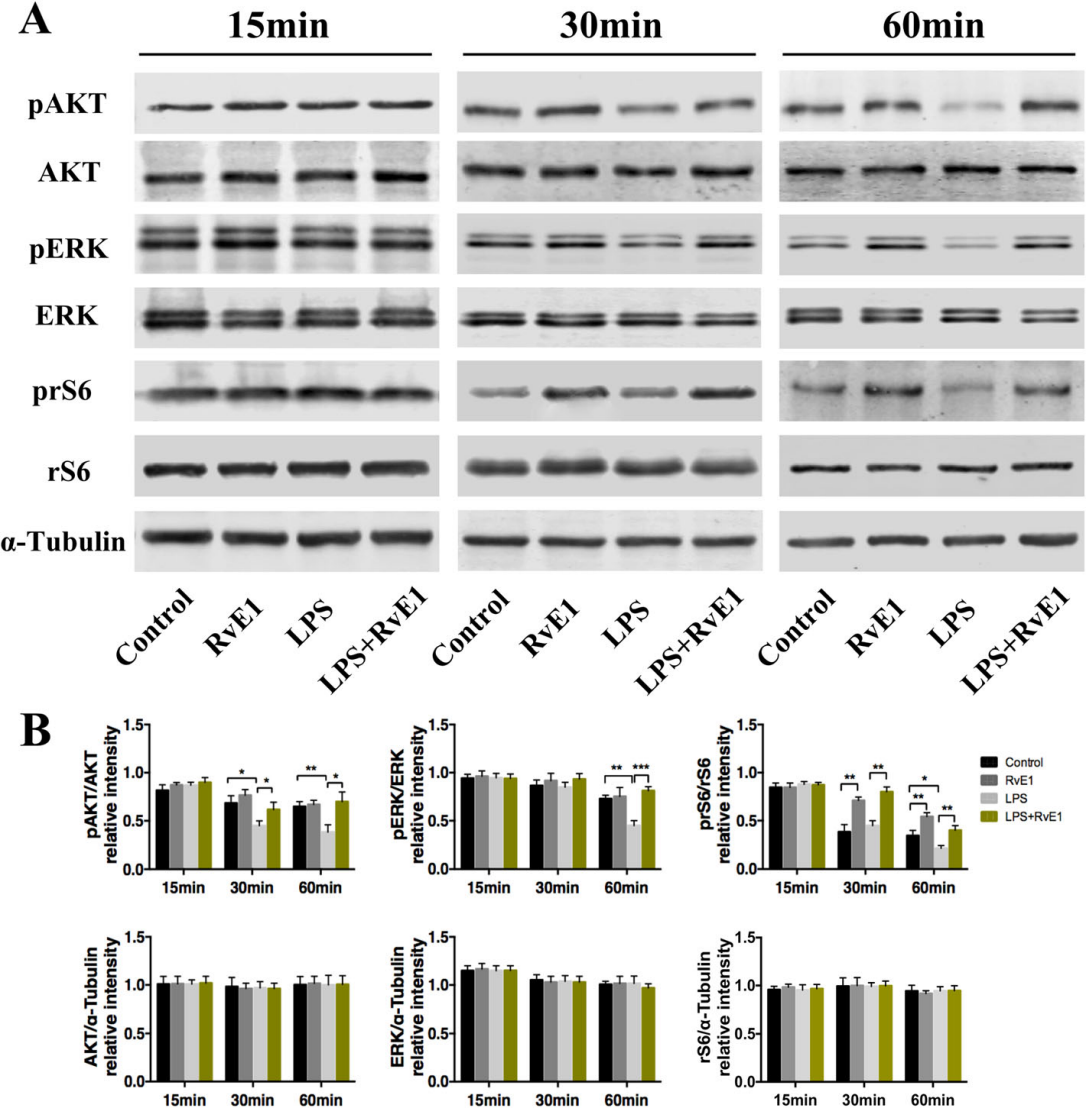
RvE1 activated PI3K-AKT and ERK signaling pathways in LPS-stimulated DPSCs. **A** The protein expression of pAKT, AKT, pERK, ERK, prS6, and rS6 were examined by Western blotting analysis. **B** Quantification of pAKT, AKT, pERK, ERK, prS6, and rS6 in 15, 30, and 60 min (**B**). *P < 0.05, **P < 0.01, ***P < 0.001. The results are mean ± standard deviation of triplicate measurements from three independent experiments

To analyze the signal transduction involved in damaged pulp repair process, we investigated the key mediators of related signaling pathways. The phosphorylated levels of AKT, ERK, and rS6 at 15, 30, and 60 min were detected. We found that the pAKT and pERK level in the RvE1 group were upregulated compared with the control group, but there was no statistical difference. The prS6 level in the RvE1 group was increased compared with the control group. While in LPS-stimulated DPSCs, the pAKT and pERK level changed significantly, especially the expression of prS6. In the LPS-induced inflammatory environment, LPS induced a decrease in the phosphorylation of AKT, ERK, and rS6 as compared to that in the negative control group; nevertheless, treatment with RvE1 could rescue this LPS-induced downregulation, especially at 30 and 60 min (*P* < 0.05) (Fig. 9A, B).

## Discussion

The maintenance of vital dental pulp following pulp injury is a matter of debate. Advanced caries or traumatic lesions can irreversibly damage the vital dental pulp to cause an excessive inflammatory response, which is not conducive to repair [30, 31]. Therefore, timely resolution of inflammation and improving the self-repair capacity of dental pulp-resident cells is particularly important. In this study, we explored the effect of RvE1 on inflammation resolution and dentin regeneration in the injured pulp tissue.

RvE1 promotes inflammation resolution in the pulp microenvironment. In this study, we found that RvE1 decreased the expression of the inflammation-related genes *TNF-α*, *IL-1β*, and *IL-6* in LPS-induced hDPSCs. We also showed that RvE1 limited the level of inflammation by decreasing the expression of the typical pro-inflammatory factors TNF-α and IL-1β in rat molar pulp tissue. Therefore, timely resolution of pulp inflammation is particularly important for preserving the vital pulp and reducing the necrosis rate of the damaged pulp. This finding was consistent with a previous finding that RvE1 exerted a protective role against pulp inflammation in infected rat dental tissue [15].

We previously reported that RvE1 can suppress the activation of DPFs to inhibit pulp inflammation, at least partially, in a ChemR23-dependent manner [17]. RvE1 reportedly has at least two receptors (ChemR23 and BLT1) [32]. Ohira et al. found that ChemR23 is expressed in ameloblasts, odontoblasts, and osteoblasts in the alveolar bone in mice. The Chemerin/ChemR23 signaling pathway affects the differentiation of ameloblasts and odontoblasts. RvE1 and Chemerin act as ChemR23 ligands. It has been speculated that RvE1 may regulate the differentiation of dental pulp mesenchymal cells via the ChemR23 receptor [33], although this was not verified. Accordingly, in this study, we found that ChemR23 was significantly expressed on hDPSCs (see Additional file 1, Fig. S3). However, the detailed role of ChemR23 in DPSCs requires further study.

RvE1 not only plays a key role in controlling inflammation, but also stimulates mineralized tissue repair. We found that RvE1 enhanced reparative dentin formation; DMP1 and DSP, which are markers of active odontoblasts that indicate vital pulp tissue integrity and function, were more strongly expressed upon RvE1 application. RvE1 promoted DPSC proliferation, chemotaxis, and differentiation. A previous study revealed an anti-inflammatory role of RvE1 in pulpitis in the short term and suggested that RvE1 may induce the formation of reparative dentine in the longer term, although this was not proven [15]. Our study showed this to indeed be the case and showed that RvE1 enhanced reparative dentin formation. We hypothesized that RvE1 regulates the inflammatory microenvironment to establish moderate inflammation, which is favorable for DPSCs to promote tissue regeneration. This is consistent with a previous finding that topically applied RvE1 is remarkably effective in inducing complete restoration of the local lesion and stimulating mineralized tissue repair in periodontitis [34]. Furthermore, the processes of osteogenesis and odontogenesis are similar, and a recent study showed that bone loss can be substantially attenuated by RvE1 treatment via increased production of osteoprotegerin by osteoblasts [35]. Moreover, lipoxin A4, another SPM, significantly enhances the proliferation, migration, and wound-healing capacity of periodontal ligament stem cells and stem cells from the apical papilla through activation of its cognate receptor, ALX/FPR2 [19]. Together, these observations provide novel evidence that RvE1 has a direct effect on the activation of inflammation-resolving pathways and stem cell restoration, thereby promoting mineralized tissue formation.

RvE1 promoted DPSC commitment in the dental mesenchyme. Pulp mesenchymal stem cells are closely associated with perivascular niches and have commonalities with pericytes [1]. Some pericytes differentiate into specialized tooth mesenchyme-derived cells (odontoblasts) during tooth growth and in response to damage in vivo [29]. One type of pericytes, NG2+ pericytes, expresses typical mesenchymal stem cell markers, including CD146, CD105, and Sca1. Upon injury, NG2+ cells are actively involved in reparative dentin formation [36]. Importantly, we found that some pulp cells were positive for CD146 and CD105 after injury, especially in the RvE1 group, whereas in the normal pulp tissue, CD146- and CD105-positive cells were only detected around the vasculature and the pulp root apex. Moreover, we detected ChemR23 expression in hDPSCs. This discovery hints that during the process of dental pulp injury, RvE1 enhances the recruitment, proliferation, and immunomodulatory functions of DPSCs located near the injury site or originating from pericytes to stimulate repair and regeneration in a ChemR23-dependent manner; however, this premise remains to be confirmed. We plan to further study the effects of RvE1 on DPSCs in the repair process by in vivo lineage tracing and evaluate the receptor-mediated mechanism.

We showed that RvE1 are likely related to the activation of the PI3K-Akt and ERK signaling pathways and the induction of rS6 phosphorylation in LPS-stimulated DPSCs. These results are consistent with those of previous studies on RvE1-ChemR23 signaling in macrophages [37]. Karim et al. demonstrated that RvE1 influences PI3K-AKT signaling by regulating multiple upstream and downstream targets, thus affecting multiple cell functions [35]. The PI3K-Akt/ERK pathways are involved in mediating cell proliferation and migration [13, 38]. rS6, a downstream target of PI3K/Akt as well as Raf/ERK signaling, is reportedly evenly and strongly expressed in differentiated and undifferentiated odontoblasts [33]. When rS6 is phosphorylated, the cells grow [37, 39]. This is the same motif by which phosphoAkt(S) Ab recognizes rS6-phosphorylated sites [40]. In this study, treatment with RvE1 markedly rescued LPS-induced downregulation of AKT, ERK, and rS6 phosphorylation events. However, further study is required to determine the specific mechanism involved in RvE1-mediated pulp repair and regeneration by using signaling pathway blockers.

## Conclusions

RvE1 reduced the necrosis rate, preserved more vital pulp, and promoted dental pulp repair and regeneration after damage. In vivo, RvE1 application promoted pulp inflammation resolution and induced effective reparative dentin reconstruction in a rat injury model. In vitro, RvE1 enhanced the proliferation, chemotaxis, and odontoblastic differentiation of hDPSCs with or without LPS, which is, at least partially, dependent on AKT, ERK, and rS6-associated signaling in the inflammatory microenvironment. Thus, RvE1 might have potential as a new therapeutic strategy to promote pulp inflammation resolution and tissue regeneration.

## Supplementary Information

**Additional file 1: Figure S1**, **Figure S2**, **Figure S3**, **Table S1**, **Table. S2**.

## Data Availability

All data generated or analyzed in this study are included in this published article [and its supplementary information files].

## Abbreviations

ALP: Alkaline phosphatase
α-MEM: α-Modified Eagle’s medium
BSP: Bone sialoprotein
CCK-8: Cell counting kit-8
DAPI: Diamidino phenylindole
DMP1: Dentin matrix protein 1
DSPP: Dentin sialophosphoprotein
DSP: Dentin sialoprotein
EDTA: Ethylene dinitrolotetra acetic acid
FBS: Fetal bovine serum
FITC: Fluorescein isothiocyanate
GAPDH: Glyceraldehyde-3-phosphate dehydrogenase
HE: Hematoxylin-eosin staining
hDPSCs: Human dental pulp stem cells
IL: Interleukin
IHC: Immunohistochemistry
LPS: Lipopolysaccharide
OD: Optical density
PBS: Phosphate buffer saline
PFA: Paraformaldehyde
RvE1: ResolvinE1
RT-PCR: Real-time quantitative PCR
RNA: Ribonucleic acid
TNF-α: Tumor necrosis factor-α
