# Supplementary material for: Resolvin E1 accelerates pulp repair by regulating inflammation and stimulating dentin regeneration in dental pulp stem cells
Source: Stem Cell Res Ther. 2021 Jan 22;12:75. doi: 10.1186/s13287-021-02141-y (PMC7821538; doi:10.1186/s13287-021-02141-y)
Supplement: Supplementary file 1 — Additional file 1: Figure S1, Figure S2, Figure S3, Table S1, Table. S2. [file 13287_2021_2141_MOESM1_ESM.docx]

**
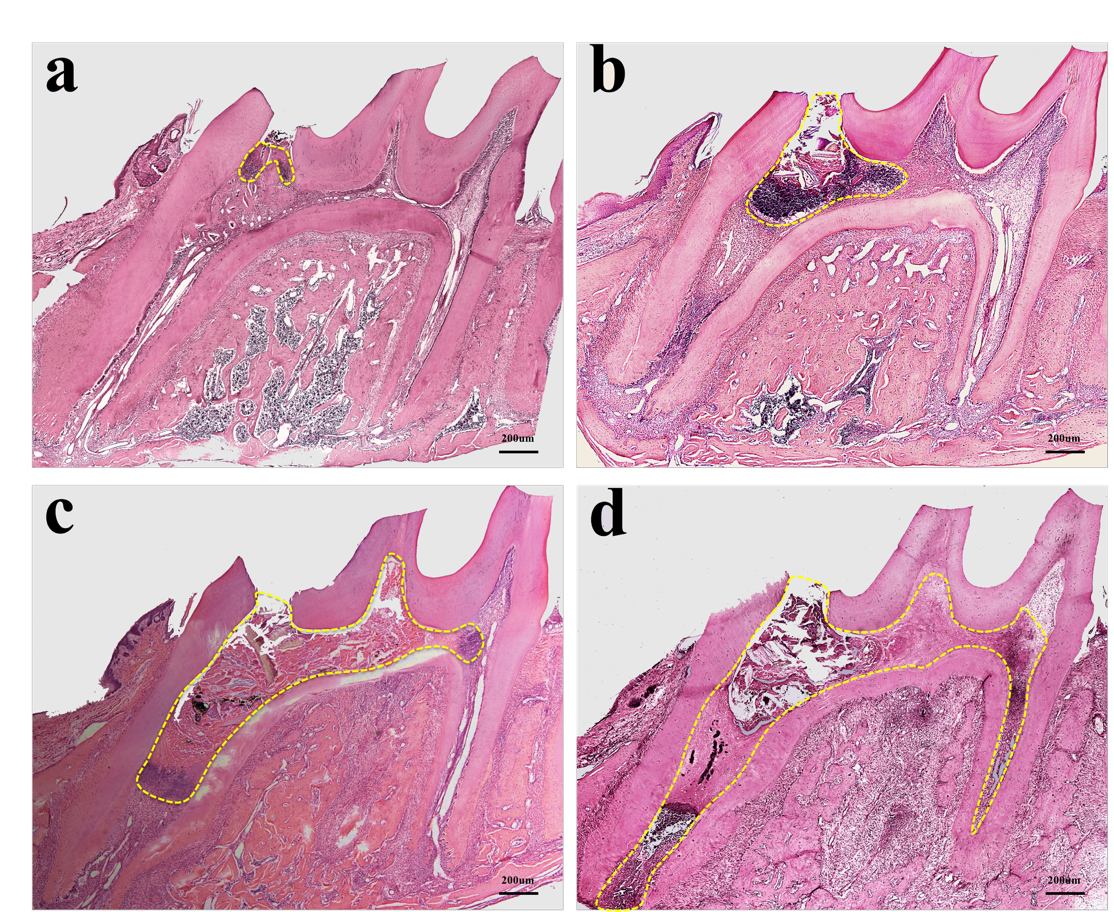
**

**Figure.S1** The process of pulp necrosis. The figure shows that in the control group, the normal structure of the necrotic pulp tissue disappears and becomes a granular, small strip or small block-like unstructured substance, and the nucleus of the necrotic tissue shrinks and nuclear fragments. The early inflammation that was not timely controlled might induce the unstable microenvironment and eventually lead to total tissue necrosis. The boundary of the necrosis area was marked by the yellow dotted line. n=12.

**Table S1**

| Time  （week） | Control | |  | RvE1 | | | Total |
| --- | --- | --- | --- | --- | --- | --- | --- |
|  | Necrosis | Repair |  | | Necrosis | Repair |  |
| 1 | 5 (50%) | 5 (50%) |  | | 3 (30%) | 7 (70%) |  |
| 2 | 6 (60%) | 4 (40%) |  | | 3 (30%) | 7 (70%) |  |
| 3 | 6 (60%) | 4 (40%) |  | | 2 (20%) | 8 (80%) |  |
| 4 | 7 (70%) | 3 (30%) |  | | 2 (20%) | 8 (80%) |  |
| Total | 24 | 16 |  | | 10 | 30 | 80 |

**Table.S1** The percentage (%) of the necrosis and healing samples in the control group and RvE1 group. About 70%-80% of the samples are repair in the RvE1 group, while in the control group, only 50% of the samples are repair at 1 week, only 30% of the samples are repair at 4 weeks.

**
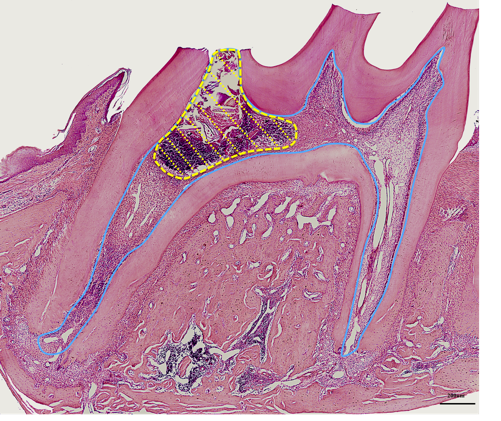
Table S2**

| Time  (week) | Necrosis Area (%) | | | | |
| --- | --- | --- | --- | --- | --- |
|  | Control | | | RvE1 | |
| 1 | | | 29% | 22% | |
| 2 | | | 40% | 18% | |
| 3 | | | 75% | 14% | |
| 4 | | | 90% | 9% | |
|  | |  | | |  |

**Table.S2** The percentage (%) of the necrotic area to total pulp area. The left figure as a diagram indicates that the yellow dotted line represents the boundary of the necrosis area. The blue line represents the whole pulp area. The right table shows that the necrosis area (%) in the control and RvE1 group. In the RvE1 group, the inflammation was resolved and the necrosis area was reduced gradually, while in the control group, the inflammation was processed fast and eventually lead to most of tissue necrosis.


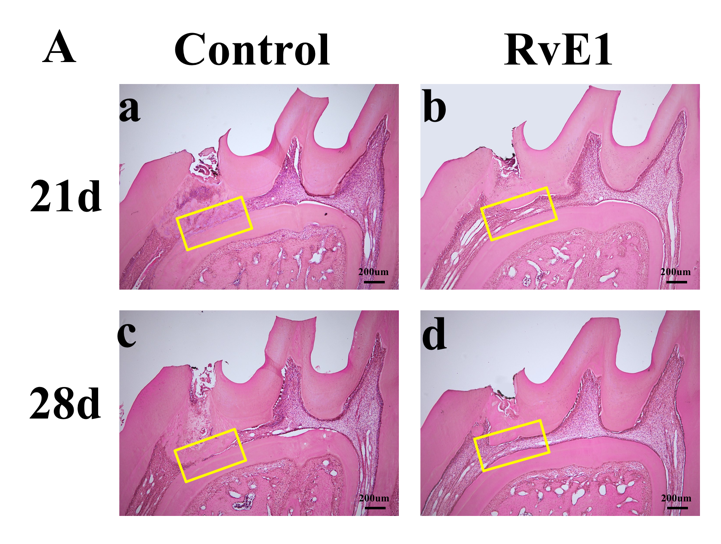


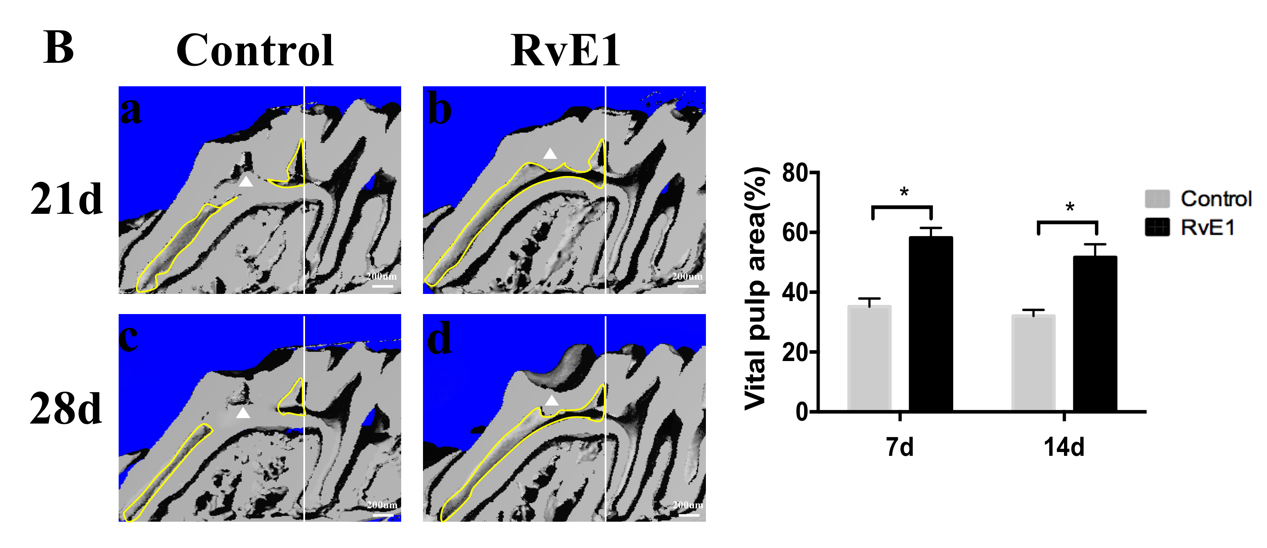


**Figure.S2** HE staining and Micro-CT to evaluate the continuity of the vital pulp and the positional relationship between the newly formed dentin bridge and the bottom of pulp chamber. A. The yellow boxes were used to observe the distance from the newly formed dentin bridge to the bottom of pulp chamber. B. The Micro-CT scan images were selected in the position where both the mesial and distal roots of the first molar penetrated from the pulp cavity to the apical foramen. We use the central cusp as the boundary (white line) to detect the remaining vital pulp area (yellow line) of the mesial pulp chamber which was more affected by the injury. The white triangle represents the reparative dentin. n=10.


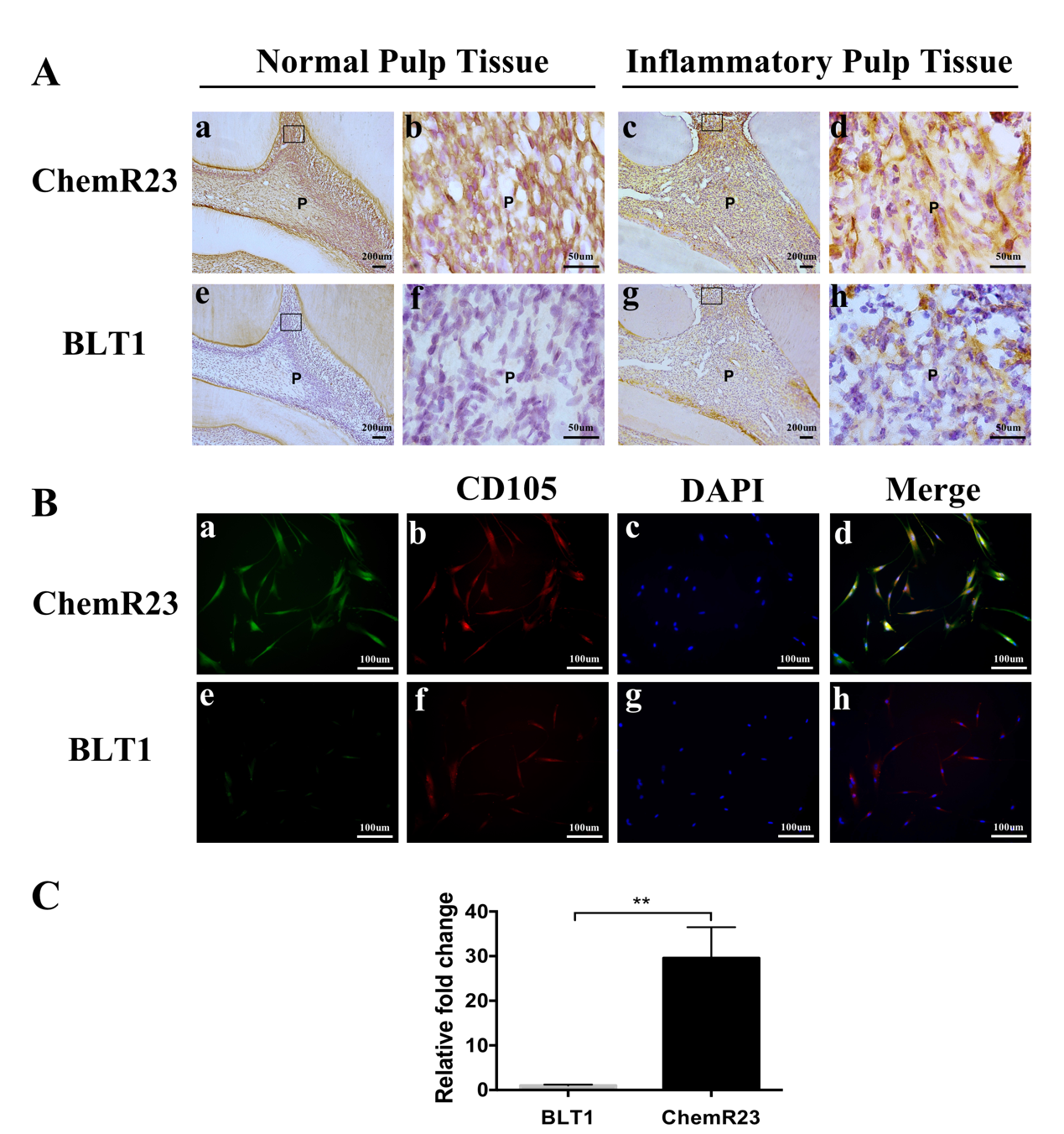


**Figure.S3** The expression of the receptors of RvE1 in rat dental pulp tissue and hDPSCs (A, B). Quantification of ChemR23 and BLT1 in hDPSCs (C). (b, d, f, h) Higher-magnification views of boxed areas in panels a, c, e, and g. P, Pulp. ***P* < 0.01. n=10. The results are mean ± standard deviation of triplicate measurements from three independent experiments.
